# Accelerated involution of germinal center in palatine tonsils in IgA nephropathy

**DOI:** 10.1371/journal.pone.0301853

**Published:** 2024-05-06

**Authors:** Hiroyuki Ueda, Kensuke Joh, Yoshimi Ueda, Hirokazu Marumoto, Masahiro Okabe, Nao Isaka, Nobuo Tsuboi, Hiromi Kojima, Yoichi Miyazaki, Takashi Yokoo

**Affiliations:** 1 Department of Internal Medicine, Division of Nephrology and Hypertension, The Jikei University School of Medicine, Tokyo, Japan; 2 Department of Pathology, The Jikei University School of Medicine, Tokyo, Japan; 3 Department of Otorhinolaryngology, The Jikei University School of Medicine, Tokyo, Japan; Universidade de Sao Paulo, BRAZIL

## Abstract

**Background:**

Altered immunological responses in the palatine tonsils may be involved in the pathogenesis of IgA nephropathy (IgAN). The germinal center serves as the site for antigen-specific humoral immune responses in the palatine tonsils. Germinal center involution is frequently observed in the palatine tonsils of IgAN (IgAN tonsils). However, the pathogenic significance of these characteristic changes remains unclear. This study aimed to investigate the morphological changes in secondary lymphoid follicles in IgAN tonsils and to evaluate the correlation between the morphometric results and the clinicopathological severity of IgAN.

**Methods:**

The tonsils of age-matched patients with recurrent tonsillitis (RT tonsils) were used as controls. The correlation between the degree of lymphoid follicular involution and histopathological severities in clinical or kidney biopsy was evaluated.

**Results:**

In total, 87 patients with IgAN were included (48% male, median age 35 years, median estimated glomerular filtration rate: 74 mL/min/1.73 m^2^). Compared to RT tonsils, IgAN tonsils showed smaller median sizes of lymphoid follicles and germinal centers (P < 0.001). The relative areas of lymphoid follicles (%LFA) and germinal centers (%GCA) in the total tonsillar tissue were smaller in the IgAN tonsils than in the RT tonsils (P < 0.001). In contrast, the median proportion of mantle zones in the total tonsillar tissue was comparable between the groups. A lower %LFA was associated with a longer period from the onset of urinary abnormalities to biopsy diagnosis and higher urinary protein excretion (P = 0.01). %LFA showed significant negative correlations with frequencies of glomeruli with both global and segmental sclerosis.

**Conclusions:**

The present study confirmed accelerated germinal center involution in the tonsils of patients with IgAN. This characteristic change in the IgAN tonsil correlates with heavy proteinuria and advanced chronic histopathological changes in the kidneys, thereby suggesting the involvement of repeated tonsillar immunoreactions during IgAN progression.

## Introduction

IgA nephropathy (IgAN) is a leading cause of primary glomerular disease worldwide. Approximately 30–40% of biopsy-diagnosed patients with IgAN progress to kidney failure despite therapies, including corticosteroids or renin-angiotensin system inhibitors [1,2]. IgAN is frequently triggered by mucosal infections, including upper respiratory tract infections, which can exacerbate urinary findings, such as hematuria and proteinuria [3]. In addition, tonsillectomy provides beneficial effects such as an earlier reduction in urinary protein excretion and protection from renal function decline in patients with IgAN. Therefore, it is highly postulated that the palatine tonsils are involved in the pathogenesis and progression of IgAN [4,5].

Palatine tonsils are a significant component of Waldeyer’s ring of lymphoid tissue. They serve as both a local mucosal defense system and the induction of a systemic immune response at the gateway of the respiratory and alimentary tracts. Morphologically and functionally, palatine tonsils comprise the following three major compartments: the crypt epithelium, interfollicular region, and secondary lymphoid follicles consisting of the mantle zone and germinal center [6]. The size of the tonsils is most prominent during childhood and is directly proportional to the bacterial load and the number of B and T cells [7]. Age-dependent reduction in size (tonsillar involution) has also been observed [8].

Tonsillar crypt epithelia exhibit a unique structure called "lymphoepithelial symbiosis," consisting of epithelial and non-epithelial cells, such as macrophages, dendritic cells, and B cells, with varying degrees of reticulation. These non-epithelial cells in crypt epithelia may participate in the uptake, transport, and presentation of antigens and contribute to antigen-specific humoral immune responses [6]. A lack of reticulation in tonsils was more frequently observed in patients with IgAN than in those with recurrent tonsillitis (RT), which is characterized by frequent episodes of acute tonsillitis without hematuria and proteinuria, and the extent of non-reticulation was correlated with the degree of renal histological damage [9]. Furthermore, some studies with small sample sizes found that germinal centers in the palatine tonsils were smaller in patients with IgAN than in those with RT [10,11]. These findings indicate that antigen-specific humoral immune responses are attenuated in IgAN tonsils, and that the degree of involution of the area related to antigen-specific humoral immune responses is associated with the progression of kidney disease.

To date, no studies have focused on the association between the degree of tonsillar secondary lymphoid follicle involution and IgAN severity. Therefore, the present study aimed to investigate the morphological changes in secondary lymphoid follicles in IgAN tonsils and to evaluate the correlation between morphometric results and the clinicopathological severity of IgAN.

## Materials and methods

### Study participants

Eighty-seven patients who were diagnosed with IgAN at our hospital between January 2009 and December 2019 and underwent tonsillectomy within 1 year of kidney biopsy were included in the study. The exclusion criteria were as follows: 1) patients aged <18 years; and 2) patients who received immunosuppressive drugs, including glucocorticoids, within 1 year before tonsillectomy. For comparison, tonsils were obtained from 27 age-matched patients with RT who did not show features of kidney function impairment and urinary abnormalities, including hematuria and proteinuria. The decision to perform tonsillectomy as a treatment for IgAN was made at the discretion of the attending physician. Patient backgrounds and laboratory findings were accessed and obtained from their medical records after approval from the Ethics Committee of the Jikei University School of Medicine (Approval date: March 9, 2020). The time from detection of urinary abnormalities to renal biopsy was defined as the period between the date when urinary protein or hematuria was first noted during regular medical checkups and the date of kidney biopsy. The estimated glomerular filtration rate (eGFR) was calculated using a modified three-variable equation for GFR in Japanese individuals: eGFR = 194 × age^-0.287^ × sCr^-1.094^ (× 0.739 if female), where sCr is the serum creatinine level.

### Immunohistochemistry

After tonsillectomy, tonsillar tissues were fixed with 4% formaldehyde and embedded in paraffin. Samples were cut into serial sections (thickness, 3 μm) for immunohistochemical analysis. After microwave antigen retrieval, sections were deparaffinized and blocked with 5% skim milk. Subsequently, the sections were incubated with the following primary antibodies: anti-HLA-DR (1:500 dilution; ab92511, Abcam, Cambridge, UK), anti-CD3 (1:50 dilution; MA5-12577, Invitrogen, South San Francisco, CA, USA), and anti-cytokeratin (ready to use; Nichirei Bioscience, Tokyo, Japan) and developed using Histofine^®^ Simple Stain MAX PO (MULTI) (Nichirei Bioscience) and a DAB staining kit (Abcam). The slides were rinsed with xylene and mounted using mounting solution and coverslips.

### Morphometric analysis

Tonsil tissue was evaluated by measuring the area of the tissue. Measurements were obtained from at least four locations containing crypts within the same tissue. The entire stained tonsillar tissue was observed under an Olympus BX53 digital virtual microscope (Olympus, Tokyo, Japan) at 40× magnification, and the images were captured. These images were analyzed using ImageJ software (National Institutes of Health, Bethesda, MA, USA). The area of each component was measured using serial sections stained with the following stains: Human Leukocyte Antigen–DR isotype (HLA-DR) for lymphoid follicles and germinal centers and cytokeratin for reticular and non-reticular crypt epithelia. Furthermore, the proportions of the lymphoid follicular area (LFA) and germinal center area to the total tonsillar tissue area were calculated by dividing the sum of the area of each region by the area of the entire measured tonsillar tissue [12]. The percentage of non-reticular crypt epithelial area was expressed relative to the total crypt epithelial area in each section, as previously reported [9].

### Kidney biopsy histopathological analysis

All kidney tissue specimens were obtained via percutaneous needle biopsy. The tissues were embedded in paraffin, cut into 3-μm sections, and stained with hematoxylin-eosin, periodic acid-Schiff, Masson’s trichrome, and periodic acid silver methenamine. All biopsy samples were stained using the following fluorescein isothiocyanate (FITC)-labeled antiantibodies (DAKO): anti-IgG, anti-IgA, anti-IgM, anti-C3, and anti-C1q. IgAN diagnosis was based on the typical histopathological features of mesangial proliferative glomerulonephritis, the presence of dominant or co-dominant glomerular IgA deposition observed using immunohistochemistry or immunofluorescence, and the presence of electron-dense mesangial deposits detected using electron microscopy. Patients with other systemic diseases associated with glomerular IgA deposition, including IgA vasculitis, liver cirrhosis, or systemic lupus erythematosus, were also excluded. We employed the Oxford classification to categorize the renal biopsy specimens, and all pathological lesions were defined according to this classification [13]. Furthermore, we determined the proportion of glomeruli with global sclerosis, segmental sclerosis, and crescentic lesions, which included cellular and fibrocellular crescents.

### Statistical analyses

The Wilcoxon–Mann–Whitney two-sample rank-sum test was used to compare non-normally distributed continuous variables between the two groups. The Jonckheere–Terpstra or Mantel–Haenszel trend test was used to analyze trends in baseline characteristics and morphological measurements among the percentages of LFA tertiles. Spearman’s rank correlation was used to determine the correlations among continuous variables between the two groups. Multiple linear regression analysis was performed to determine the association between independent variables. Statistical significance was set at P < 0.05. All statistical analyses were performed using R software (version 2.4 patched) and GraphPad Prism 8.0 (GraphPad Software, San Diego, CA, USA).

### Study approval

This study was approved by the Ethics Committee of the Jikei University School of Medicine (Approval No: 31–431 [10013]). As this was a retrospective study, information on the research plan was posted online on our university/hospital website, patients were allowed to refuse participation, and individual informed consent was not required. The participants were identified using numbers rather than names, and all data were anonymized before we accessed them. This study adhered to the principles of the Declaration of Helsinki. All methods were performed in accordance with the institutional guidelines of the ethics committee of The Jikei University School of Medicine (Tokyo, Japan).

### Data availability

The datasets generated and analyzed during the current study are available from the corresponding author upon reasonable request.

## Results

### Morphometric evaluation of size and proportion of lymphoid follicles and their significant components in IgAN tonsils

**Fig 1:**
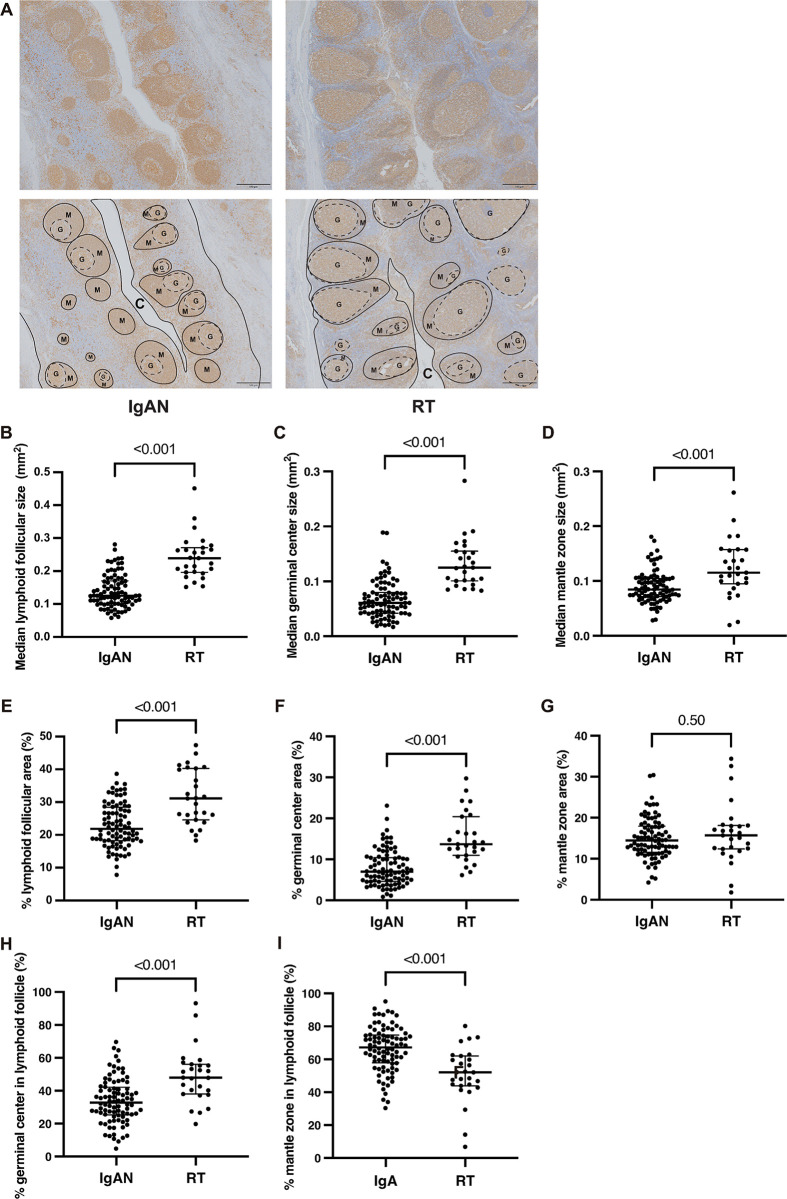
Comparison of the size and proportion of secondary lymphoid follicles between IgAN and RT tonsils. **(A)** Representative images of IgAN (right panels) and RT (left panels) tonsils stained with an anti-HLA-DR antibody. Lower panels illustrate secondary lymphoid follicles indicated with solid lines, mantle zones are marked with the letter “M,” and germinal centers are indicated using dotted circles and marked with “G.” The letter “C” refers to the crypt. Magnification, 40×; scale bar, 500 μm. **(B–D)** Comparison of the size distribution of lymphoid follicles (B), germinal centers (C), and mantle zone (D) between IgAN (N = 87) and RT (N = 27) tonsils. The median sizes of lymphoid follicles (B), germinal centers (C), and mantle zones (D) in IgAN tonsils were smaller than those in RT tonsils (P < 0.001). **(E–G)** Comparison of the percentages of lymphoid follicles (% lymphoid follicular area, E), germinal centers (% germinal center area, F), and mantle zones (% mantle zone area, G) in the total tonsillar tissue of the IgAN and RT tonsils. The median % lymphoid follicular area (E) and % germinal center area (F) tonsils were smaller in the IgAN tonsils than in the RT tonsils (P < 0.001). In contrast, the median % mantle zone area (G) was comparable between the IgA and RT tonsils. **(H and I)** Comparison of the percentages of germinal center area (H) and mantle zone area (I) in the lymphoid follicles of IgAN and RT tonsils. Lymphoid follicles were accompanied by smaller percentages of germinal center area (H) and larger percentages of mantle zone area (I) in IgAN tonsils than in RT tonsils (P < 0.001).

First, we assessed the size and proportion of lymphoid follicles and their significant components, i.e., germinal centers and mantle zones, in the tonsils of patients with IgAN (IgAN tonsils; N = 87) and compared them with age-matched patients with recurrent tonsillitis (RT tonsils; N = 27) (**Fig 1**). The sizes of the secondary lymphoid follicles in the IgAN tonsils were more heterogeneous than those in the RT tonsils (**Fig 1A**). The proportions of germinal centers in lymphoid follicles were smaller in the IgAN tonsils than in the RT tonsils. The median size of lymphoid follicles (**Fig 1B**), germinal centers (**Fig 1C**), and mantle zones (**Fig 1D**) were significantly smaller in the IgAN tonsils than in the RT tonsils (P < 0.001). Similarly, the median proportion of lymphoid follicles (% lymphoid follicular area [%LFA], **Fig 1E**) and germinal centers (% germinal center area, **Fig 1F**) in the total tonsillar tissue was significantly smaller in the IgAN tonsils than in the RT tonsils (P < 0.001). In contrast, the median proportions of mantle zones in the total tonsillar tissue (% mantle zone area, **Fig 1G**) were comparable between IgA and RT tonsils. Furthermore, in comparison with those in the RT tonsils, lymphoid follicles in the IgAN tonsils were associated with smaller proportions of germinal centers (**Fig 1H**) and were notably dominated by the mantle zone (**Fig 1I**).

We also assessed the extent of the non-reticular area in the cytokeratin-positive crypt epithelia in the IgAN tonsils (**S1A Fig**). The median percentage of the non-reticular area in the total crypt epithelial area (% non-reticular area) in the IgAN tonsils was larger than that in the RT tonsils (**S1B Fig**). Furthermore, in the IgAN tonsils, the % non-reticular area showed a significant negative correlation with %LFA (**S1C Fig)** and % germinal center area (**S1D Fig**). For subsequent clinicopathological analysis, %LFA was used because lymphoid follicles in IgAN tonsils frequently lack a germinal center on the section.

### Comparison of clinical characteristics among IgAN patient groups categorized according to the %LFA of the tonsils

To clarify the clinical characteristics of patients with IgAN related to the degree of involution of lymphoid follicles in IgAN tonsils, we categorized patients with IgAN into tertile groups according to their %LFA levels as follows: high (26.0–38.6%), intermediate (19.0–25.6%), and low (7.8–18.9%). When considering the patient characteristics at the time of renal biopsy, sex, age, and body mass index were comparable among the tertile groups based on the %LFA (**Table 1**). Similarly, a history of smoking, recurrent tonsillitis, and macroscopic hematuria did not differ significantly among the %LFA groups. However, the median time from the onset of urinary abnormalities to diagnostic renal biopsy was longer in the lower %LFA tertiles than in the higher %LFA tertiles (P = 0.02). Furthermore, the number of patients with hypertension at the time of diagnostic kidney biopsy was higher in the lower %LFA tertile than in the higher %LFA tertile (P = 0.03). Laboratory findings at diagnostic kidney biopsy revealed that median urinary protein excretion increased inversely with tertiles of %LFA (P = 0.01); however, the severity of hematuria, serum creatinine, and estimated glomerular filtration rate (eGFR) levels were not statistically different among the tertiles of %LFA.

**Table 1:** Differences in baseline characteristics in patients with IgAN categorized into tertiles according to percentages of lymphoid follicular area.

	All	%LFA			P-value
		High	Intermediate	Low	
		(26.0–38.6)	(19.0–25.6)	(7.8–18.9)	
N	87	29	29	29	
Female sex, N (%)	46 (52)	15 (52)	14 (48)	17 (59)	0.59
Age at tonsillectomy	35 [30–41]	33 [25–38]	37 [33–42]	33 [30–45]	0.54
BMI	20 [19–22]	21 [19–24]	21.3 [20–23]	21.0 [19–22]	0.98
History of smoking, N (%)	19 (22)	6 (21)	7 (24)	6 (21)	0.99
History of RT, N (%)	13 (14)	6 (21)	4 (14)	3 (10)	0.52
History of macroscopic hematuria, N (%)	22 (24)	13 (45)	4 (14)	4 (14)	0.11
Time from urinary abnormalities to renal biopsy, year	3 [1.2–10]	1.9 [0.5–4.1]	6.2 [2–14.8]	3.8 [1.6–13.3]	0.02*
Hypertension, N (%)	28 (35)	5 (17)	12 (41)	11 (38)	0.03*
Cr (mg/dL)	0.86 [0.67–1.07]	0.78 [0.65–1.00]	0.9 [0.66–1.16]	0.93 [0.69–1.14]	0.19
eGFR (mL/min/1.73m^2^)	74 [61–87]	81 [7–92]	73 [53–85]	70 [56–84]	0.05
IgA (mg/dL)	317 [255–392]	284 [236–372]	351 [271–435]	321 [251–383]	0.55
C3	97 [83–114]	97 [76–116]	98 [80–113]	102 [85–117]	0.29
IgA/C3	3.1 [2.4–4.1]	2.4 [1.2–3.1]	2.5 [1.4–3.6]	2.2 [1.2–2.9]	0.55
UPE (g/day)	0.68 [0.51–1.18]	0.56 [0.39–0.86]	0.64 [0.38–1.15]	0.92 [0.65–1.59]	0.01*
Hematuria, RBC/HPF					
< 20/HPF, N (%)	35 (40)	11 (38)	12 (42)	12 (42)	0.78
20–99, N (%)	43 (50)	15 (52)	14 (48)	14 (48)	0.79
≥ 100/HPF, N (%)	9 (10)	3 (10)	3 (10)	3 (10)	0.99

Abbreviations: BMI, body mass index; eGFR, estimated glomerular filtration; HPF, high power field; IgAN IgA nephropathy; %LFA, percentages of lymphoid follicular areas in total tonsillar tissue; RBC, red blood cells; RT, recurrent tonsillitis; UPE, urinary protein excretion.

* Statistically significant.

where, N represents the number of patients. Other values are expressed as mean values with standard deviations or median values with interquartile ranges.

### Comparison of kidney biopsy histopathological findings among IgAN patient groups categorized according to the %LFA of the tonsils

A summary of the renal biopsy findings in relation to %LFA is presented in **Table 2**. According to the Oxford classification, the proportion of patients with S1 and C1+2 increased inversely with tertiles of %LFA. In contrast, the proportion of patients with M1, E1, or T1+2 did not differ among the tertiles of %LFA.

**Table 2:** Comparisons of kidney pathologies according to tertiles of percentages of lymphoid follicular area.

	All		%LFA		
		High	Intermediate	Low	P-value
		(26.0–38.6)	(19.0–25.6)	(7.8–18.9)	
N	87	29	29	29	
**Oxford Classification**					
M1, N (%)	6 (6)	2 (7)	2 (7)	2 (7)	0.99
E1, N (%)	54 (62)	18 (62)	16 (55)	20 (69)	0.58
S1, N (%)	68 (78)	18 (62)	23 (79)	27 (93)	0.004*
T1+2, N (%)	10 (11)	1 (3)	6 (21)	3 (10.3)	0.41
C1+2, N (%)	47 (54)	10 (34)	16 (55)	21 (72)	0.003*

Abbreviations: %LFA Percentages of lymphoid follicular areas in total tonsillar tissue.

* Statistically significant.

where, N represents the number of patients.

**Fig 2:**
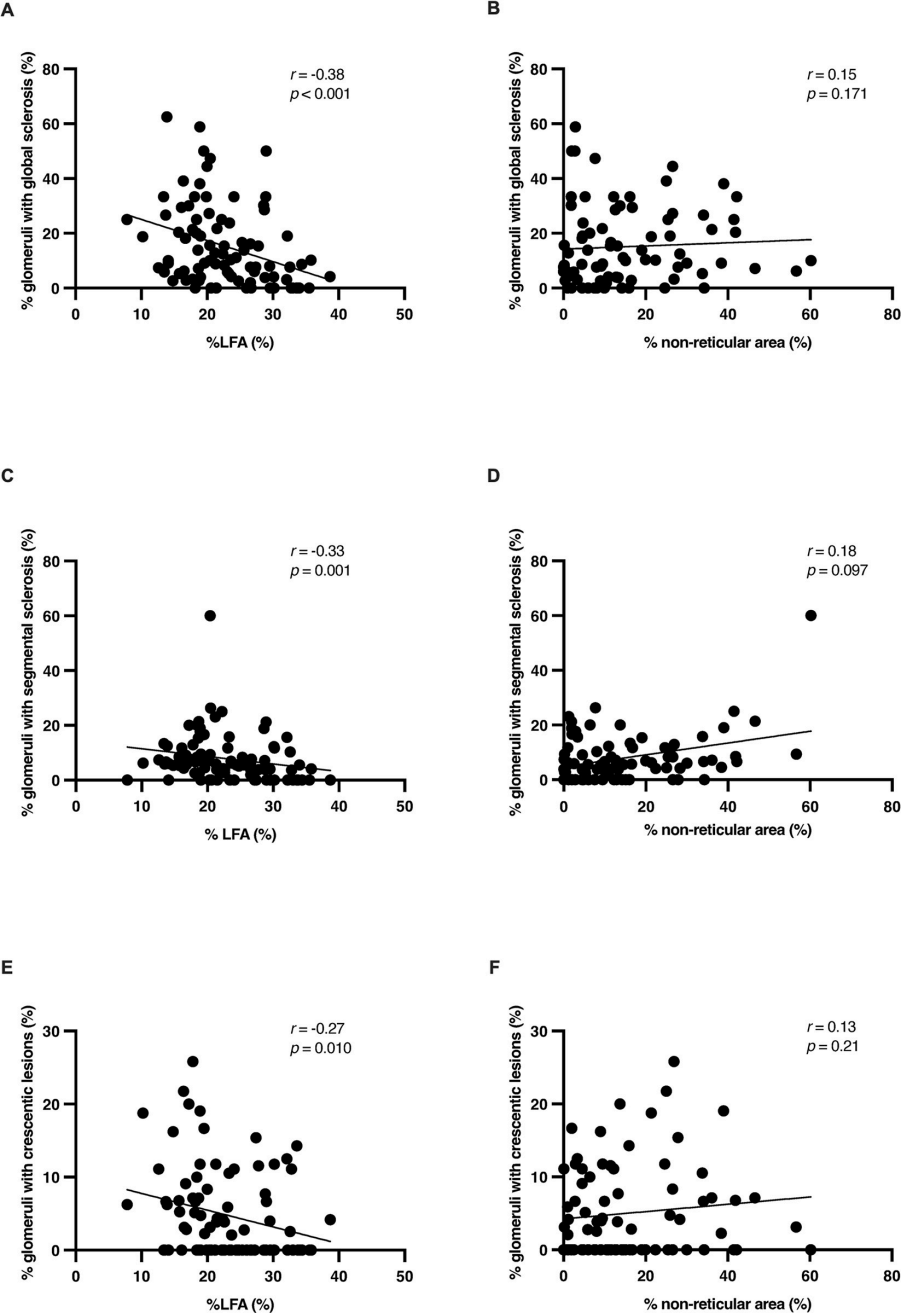
Correlation between tonsillar morphological changes, i.e., %LFA and % non-reticular area, and percentage of glomeruli affected in patients with IgAN. Correlation between (A) %LFA and % glomeruli with global sclerosis, (B) % non-reticular area and % glomeruli with global sclerosis, (C) %LFA and % glomeruli with segmental sclerosis, (D) % non-reticular area and % glomeruli with segmental sclerosis, (E) %LFA and % glomeruli with crescentic lesions, and (F) % non-reticular area and % glomeruli with crescentic lesions. Tonsillar %LFA was negatively correlated with the percentage of glomeruli with global sclerosis (**A**) (*r* = –0.38, P < 0.001), segmental sclerosis (C) (*r* = –0.33, P = 0.001), and crescentic lesions (**E**) (*r* = –0.27, P = 0.010). In contrast, % non-reticular area did not show a significant correlation with the percentage of glomeruli affected. Correlations were tested using nonparametric Spearman’s correlation coefficient.

We also assessed the correlation between the percentage of glomeruli showing global sclerosis, segmental sclerosis, or crescentic lesions (cellular or fibrocellular crescent) and %LFA or % non-reticular area (**Fig 2**). The frequency of glomeruli showing global sclerosis (**Fig 2A**), and segmental sclerosis (**Fig 2C**) displayed a significant negative correlation with %LFA (global sclerosis vs. %LFA, *r* = –0.38, P < 0.001; segmental sclerosis vs. %LFA, *r* = –0.33, P = 0.001). Furthermore, the frequency of glomeruli with crescentic lesions showed a weak negative correlation with %LFA (**Fig 2E**, *r* = –0.27, P = 0.010). In contrast, % non-reticular area showed no significant correlation with the percentage of glomeruli in any of the lesions (**Fig 2B, 2D and 2F**). After logarithmic transformation of the percentages of glomeruli with global and segmental sclerosis, age, and the median time from the onset of urinary abnormalities to kidney biopsy, a multiple linear regression analysis was performed to determine the factors associated with the proportion of sclerosed glomeruli (**Table 3**). Owing to the significant interaction between sex and time from the onset of urinary abnormalities to kidney biopsy in the multiple regression analysis, we performed separate analyses for each group. In the female group, the percentage of glomeruli with sclerotic lesions increased with hypertension and %LFA. In the male group, the percentage of sclerosed glomeruli increased with time from the onset of urinary abnormalities to kidney biopsy and %LFA. Overall, %LFA was an independent factor associated with the percentage of sclerosed glomeruli in both sex groups.

**Table 3:** Comparisons of kidney pathologies according to tertiles of the percentages of lymphoid follicular areas.

	β Coefficient	95% CI	p-value
**Female**			
Intercept	1.25	−2.84–−5.35	0.54
Age	0.68	−0.45–1.81	0.23
Hypertension	0.86	0.24–1.49	0.007*
Time from urinary abnormalities to renal biopsy	0.023	−0.16–0.21	0.80
%LFA	−0.057	−0.09–−0.01	0.006*
**Male**			
Intercept	0.33	−3.48–4.16	0.85
Age	0.73	−0.30–1.77	0.159
Hypertension	−0.049	−0.70–0.60	0.87
Time from urinary abnormalities to renal biopsy	0.37	0.19–0.55	<0.001*
%LFA	−0.05	−0.09–−0.008	0.018*

95% CI, confidence interval; %LFA, percentage of lymphoid follicular areas in total tonsillar tissue.

* Statistically significant.

## Discussion

In this cross-sectional study, we showed that reductions in the size of lymphoid follicles and %LFA were more common in IgAN tonsils than in RT tonsils and that these histological changes in IgAN tonsils were mainly due to the reduction in the size of the germinal centers. Patients with IgAN with lower %LFA had longer durations of urinary abnormalities and more severe proteinuria at the time of diagnostic kidney biopsy. Furthermore, %LFA showed significant negative correlations with the frequency of glomeruli with both global and segmental sclerosis. These findings suggested that the accelerated involution of the germinal center in the tonsils and its degree were correlated with chronic progression of kidney disease. This indicates that the accelerated involution of the germinal center in the tonsils is critically involved in the pathogenesis and progression of IgAN.

In this study, we first demonstrated that IgAN tonsils consist of smaller lymphoid follicles comprising smaller germinal centers and mantle zones than RT tonsils. These findings are consistent with those of previous studies with small sample sizes [10,11]. Our analysis revealed that the % mantle zone area was comparable between the IgAN and RT tonsils. Thus, the smaller lymphoid follicles and lower %LFA in IgAN tonsils were mainly attributed to the reduction in the size of germinal centers compared with RT tonsils. Due to the lack of tonsils from healthy controls, we cannot conclude that these findings are characteristic of IgAN tonsils but not of tonsils from healthy subjects without tonsillar diseases. However, a previous report showed that the mean % germinal center area and % mantle zone area were lower in RT tonsils than in age-matched tonsils from patients without tonsillar disease [14], indicating that germinal center involution is a prominent feature of IgAN tonsils.

Next, we revealed that compared to patients with IgAN with higher %LFA, those with IgAN with lower %LFA had a longer period of urinary abnormalities and higher frequencies of hypertension, along with higher urinary protein excretion. Additionally, the %LFA was negatively correlated with the percentage of glomeruli with global or segmental sclerosis. Another cohort study on the beneficial effect of tonsillectomy for IgAN recurrence after kidney transplantation reported that patients with IgAN who underwent tonsillectomy after kidney transplantation had highly involuted germinal centers [15]. These findings suggest that the involution of the germinal center is associated with chronic progression of glomerular disease.

The germinal center is a compartment of secondary lymphoid follicles where T cell-dependent antibody responses occur, resulting in affinity maturation, isotype switching, and the generation of long-lived plasma cells and memory B cells. The mantle zone is mainly composed of recirculating naïve B cells. Aging potentially affects the immunocyte profile of the palatine tonsils. The total B-cell population and size of lymphoid follicles decrease after puberty as the ratio of T to B cells increases [16–18]. Furthermore, the proportion of germinal center B cells in the total B cell population of the tonsil decreases with age, whereas the proportion of naïve B cells increases with age [19]. Thus, our observations of IgAN tonsils are similar to age-related changes in tonsils. Our study found that the median age of patients with IgAN was comparable between tertiles of %LFA. Furthermore, the %LFA was an independent predictor of the percentage of sclerosed glomeruli after adjusting for age. Another potential factor accelerating the involution of the germinal center in the IgAN tonsils is recurrent inflammation, which may influence the immunocyte profile in the palatine tonsils. In patients with RT tonsils, the density of immunoglobulin-producing B cells in germinal centers and extra-follicular areas is markedly and progressively reduced at an approximate age of >10 years [20]. However, in our study, most patients with IgAN had no history of clinically apparent recurrent tonsillitis. Recently, overexpression of a proliferation-inducing ligand (APRIL) and Toll-like receptors (TLRs) in nasopharynx-associated lymphoid tissue (NALT) in patients and rodent models with IgAN has been reported and speculated to be involved in pathogenesis [21–25]. Germinal centers in IgAN tonsils contain B cells that aberrantly express APRIL, which may be prone to the synthesis of galactose-deficient IgA1 in a T cell-independent manner [21,26]. Aberrant expression of APRIL in IgAN germinal centers can accelerate B cell apoptosis in the germinal center because receptor, transmembrane activator, and cyclophilin ligand interactor (TACI) signaling can induce this apoptosis [27,28]. In addition, persistent activation of the innate immune system via the aberrant expression of TRLs may accelerate the involution of reticulation lymphoepithelial symbiosis in crypt epithelia and attenuate antigen-specific humoral immune responses. We observed that the lack of reticulation in crypt epithelia was more severe in the IgAN tonsils than in the RT tonsils, as previously reported. Furthermore, % non-reticular area was inversely correlated with %LFA and % germinal center area, whereas a direct correlation was not observed between % non-reticular area and the proportion of sclerosed glomeruli. Notably, most of these tonsillar morphological changes insidiously proceed with chronic progression of kidney disease.

The major limitation of this study was that it was cross-sectional and morphological; we failed to establish an actual cause-and-effect relationship or address the underlying mechanism owing to the study’s cross-sectional design. Additionally, all patients included in the study were Japanese; therefore, the findings of these studies may not be generalizable to patients from other regions or countries. Despite these limitations, this study adds to our understanding of the importance of tonsillar microenvironment in IgAN pathogenesis.

In conclusion, the present study showed that the degree of involution of secondary lymphoid follicles in IgAN tonsils is significantly correlated with the severity of proteinuria and the renal histopathology. These findings suggest that abnormal immune responses in the palatine tonsils are closely associated with IgAN progression. Further studies are required to clarify the underlying mechanism of the NALT–kidney axis in IgAN.

## Supporting information

S1 Fig Lack of reticulation in crypt epithelia is more common in IgAN tonsils than in RT tonsils, and the percentage of non-reticular areas is negatively correlated with % lymphoid follicular area and % germinal center area. **(A)** Immunostaining with cytokeratin demonstrates crypt epithelia in the IgAN (right panel) and RT (left panel) tonsils. Lack of reticulation (arrow) in crypt epithelia is more frequently observed in IgAN tonsils than in RT tonsils. Magnification: 40×; scale: 500 μm. **(B)** The median percentage of non-reticular area in the crypt epithelia (% non-reticular area) was higher in the IgAN tonsils than in the RT tonsils (P < 0.001). **(C and D)** % non-reticular area was inversely correlated with % lymphoid follicular area (C) (*r* = − 0.35, P = 0.001) and % germinal center area (D) (*r* = − 0.26, P = 0.015).
(TIF)

S1 File Dataset. (CSV)

S1 Dataset (CSV)

## References

[1] Koyama A, Igarashi M, Kobayashi M. Natural history and risk factors for immunoglobulin a nephropathy in Japan. Am J Kidney Dis. 1997;29: 526–532. 9100040 10.1016/s0272-6386(97)90333-4

[2] Alamartine E, Sabatier J-C, Guerin C, Berliet J-M, Berthoux F. Prognostic Factors in Mesangial IgA Glomerulonephritis: An Extensive Study With Univariate and Multivariate Analyses. Am J Kidney Dis. 1991;18: 12–19. doi: 10.1016/s0272-6386(12)80284-8 2063844

[3] D’Amico G. Clinical features and natural history in adults with IgA nephropathy. Am J Kidney Dis. 1988;12: 353–357. doi: 10.1016/s0272-6386(88)80023-4 3055956

[4] Kawamura T, Yoshimura M, Miyazaki Y, Okamoto H, Kimura K, Hirano K, et al. A multicenter randomized controlled trial of tonsillectomy combined with steroid pulse therapy in patients with immunoglobulin A nephropathy. Nephrol Dial Transplant. 2014;29: 1546–1553. doi: 10.1093/ndt/gfu020 24596084 PMC4106640

[5] Hirano K, Matsuzaki K, Yasuda T, Nishikawa M, Yasuda Y, Koike K, et al. Association Between Tonsillectomy and Outcomes in Patients With Immunoglobulin A Nephropathy. JAMA Netw Open. 2019;2: e194772. doi: 10.1001/jamanetworkopen.2019.4772 31150076 PMC6547111

[6] Brandtzaeg P. Chapter 103—Immunobiology of the Tonsils and Adenoids. In: Mestecky J, Strober W, Russell MW, Kelsall BL, Cheroutre H, Lambrecht BN, editors. Mucosal Immunology (Fourth Edition). Boston: Academic Press; 2015. pp. 1985–2016.

[7] Siegel G, Linse R, Macheleidt S. Factors of tonsillar involution: age-dependent changes in B-cell activation and Langerhans’ cell density. Arch Otorhinolaryngol. 1982;236: 261–269. doi: 10.1007/BF00454218 6984327

[8] Jung KY, Lim HH, Choi G, Choi JO. Age-related changes of IgA immunocytes and serum and salivary IgA after tonsillectomy. Acta Otolaryngol Suppl. 1996;523: 115–119. 9082753

[9] Sato Y, Hotta O, Taguma Y, Takasaka T, Nose M. IgA nephropathy with poorly developed lymphoepithelial symbiosis of the palatine tonsils. Nephron. 1996;74: 301–308. doi: 10.1159/000189325 8893145

[10] Adachi M, Sato M, Miyazaki M, Hotta O, Hozawa K, Sato T, et al. Steroid pulse therapy transiently destroys the discriminative histological structure of tonsils in IgA nephropathy: Tonsillectomy should be performed before or just after steroid pulse therapy. Auris Nasus Larynx. 2018;45: 1206–1213. doi: 10.1016/j.anl.2018.04.009 29789195

[11] Takechi H, Oda T, Hotta O, Yamamoto K, Oshima N, Matsunobu T, et al. Clinical and immunological implications of increase in CD208+ dendritic cells in tonsils of patients with immunoglobulin A nephropathy. Nephrol Dial Transplant. 2013;28: 3004–3013. doi: 10.1093/ndt/gft399 24081865 PMC3843345

[12] Brandtzaeg P, Surjan L Jr, Berdal P. Immunoglobulin systems of human tonsils. I. Control subjects of various ages: quantification of Ig-producing cells, tonsillar morphometry and serum Ig concentrations. Clin Exp Immunol. 1978;31: 367–381. 350457 PMC1541246

[13] Working Group of the International IgA Nephropathy Network and the Renal Pathology Society, Roberts ISD, Cook HT, Troyanov S, Alpers CE, Amore A, et al. The Oxford classification of IgA nephropathy: pathology definitions, correlations, and reproducibility. Kidney Int. 2009;76: 546–556. doi: 10.1038/ki.2009.168 19571790

[14] Korsrud FR, Brandtzaeg P. Immune systems of human nasopharyngeal and palatine tonsils: histomorphometry of lymphoid components and quantification of immunoglobulin-producing cells in health and disease. Clin Exp Immunol. 1980;39: 361–370. 6993071 PMC1538067

[15] Kawabe M, Yamamoto I, Yamakawa T, Katsumata H, Isaka N, Katsuma A, et al. Association Between Galactose-Deficient IgA1 Derived From the Tonsils and Recurrence of IgA Nephropathy in Patients Who Underwent Kidney Transplantation. Front Immunol. 2020;11: 2068. doi: 10.3389/fimmu.2020.02068 33013875 PMC7494805

[16] Wiatrak BJ, Woolley AL. Pharyngitis and adenotonsillar disease. Otolaryngology Head and Neck Surgery, ed. 2005;3: 188–215.

[17] Tajima K. Age changes in the human palatine tonsils, with remarks on the histology of the secondary nodules. Arch Histol Jpn. 1967;28: 63–78. doi: 10.1679/aohc1950.28.63 6067856

[18] Harada K. [The histopathological study of human palatine tonsils—especially age changes]. Nihon Jibiinkoka Gakkai Kaiho. 1989;92: 1049–1064. doi: 10.3950/jibiinkoka.92.1049 2809872

[19] Kolar GR, Mehta D, Wilson PC, Capra JD. Diversity of the Ig repertoire is maintained with age in spite of reduced germinal centre cells in human tonsil lymphoid tissue. Scand J Immunol. 2006;64: 314–324. doi: 10.1111/j.1365-3083.2006.01817.x 16918701

[20] Surjan L Jr, Brandtzaeg P, Berdal P. Immunoglobulin systems of human tonsils. II. Patients with chronic tonsillitis or tonsillar hyperplasia: quantification of Ig-producing cells, tonsillar morphometry and serum Ig concentrations. Clin Exp Immunol. 1978;31: 382–390. 350458 PMC1541256

[21] Muto M, Manfroi B, Suzuki H, Joh K, Nagai M, Wakai S, et al. Toll-Like Receptor 9 Stimulation Induces Aberrant Expression of a Proliferation-Inducing Ligand by Tonsillar Germinal Center B Cells in IgA Nephropathy. J Am Soc Nephrol. 2017;28: 1227–1238. doi: 10.1681/ASN.2016050496 27920152 PMC5373453

[22] Kano T, Suzuki H, Makita Y, Fukao Y, Suzuki Y. Nasal-associated lymphoid tissue is the major induction site for nephritogenic IgA in murine IgA nephropathy. Kidney Int. 2021;100: 364–376. doi: 10.1016/j.kint.2021.04.026 33961870

[23] Suzuki H, Suzuki Y, Narita I, Aizawa M, Kihara M, Yamanaka T, et al. Toll-like receptor 9 affects severity of IgA nephropathy. J Am Soc Nephrol. 2008;19: 2384–2395. doi: 10.1681/ASN.2007121311 18776126 PMC2588104

[24] Sato D, Suzuki Y, Kano T, Suzuki H, Matsuoka J, Yokoi H, et al. Tonsillar TLR9 expression and efficacy of tonsillectomy with steroid pulse therapy in IgA nephropathy patients. Nephrol Dial Transplant. 2012;27: 1090–1097. doi: 10.1093/ndt/gfr403 21778277

[25] Goto T, Bandoh N, Yoshizaki T, Nozawa H, Takahara M, Ueda S, et al. Increase in B-cell-activation factor (BAFF) and IFN-gamma productions by tonsillar mononuclear cells stimulated with deoxycytidyl-deoxyguanosine oligodeoxynucleotides (CpG-ODN) in patients with IgA nephropathy. Clin Immunol. 2008;126: 260–269. doi: 10.1016/j.clim.2007.11.003 18249037

[26] Yamaguchi H, Goto S, Takahashi N, Tsuchida M, Watanabe H, Yamamoto S, et al. Aberrant mucosal immunoreaction to tonsillar microbiota in immunoglobulin A nephropathy. Nephrol Dial Transplant. 2021;36: 75–86. doi: 10.1093/ndt/gfaa223 33099625 PMC7771982

[27] Tsuji S, Stein L, Kamada N, Nuñez G, Bram R, Vallance BA, et al. TACI deficiency enhances antibody avidity and clearance of an intestinal pathogen. J Clin Invest. 2014;124: 4857–4866. doi: 10.1172/JCI74428 25271628 PMC4347253

[28] Yan M, Wang H, Chan B, Roose-Girma M, Erickson S, Baker T, et al. Activation and accumulation of B cells in TACI-deficient mice. Nat Immunol. 2001;2: 638–643. doi: 10.1038/89790 11429549

