# Supplementary figures and images for: Accelerated involution of germinal center in palatine tonsils in IgA nephropathy
Source: PLoS One. 2024 May 6;19(5):e0301853. doi: 10.1371/journal.pone.0301853 (PMC11073668; doi:10.1371/journal.pone.0301853)

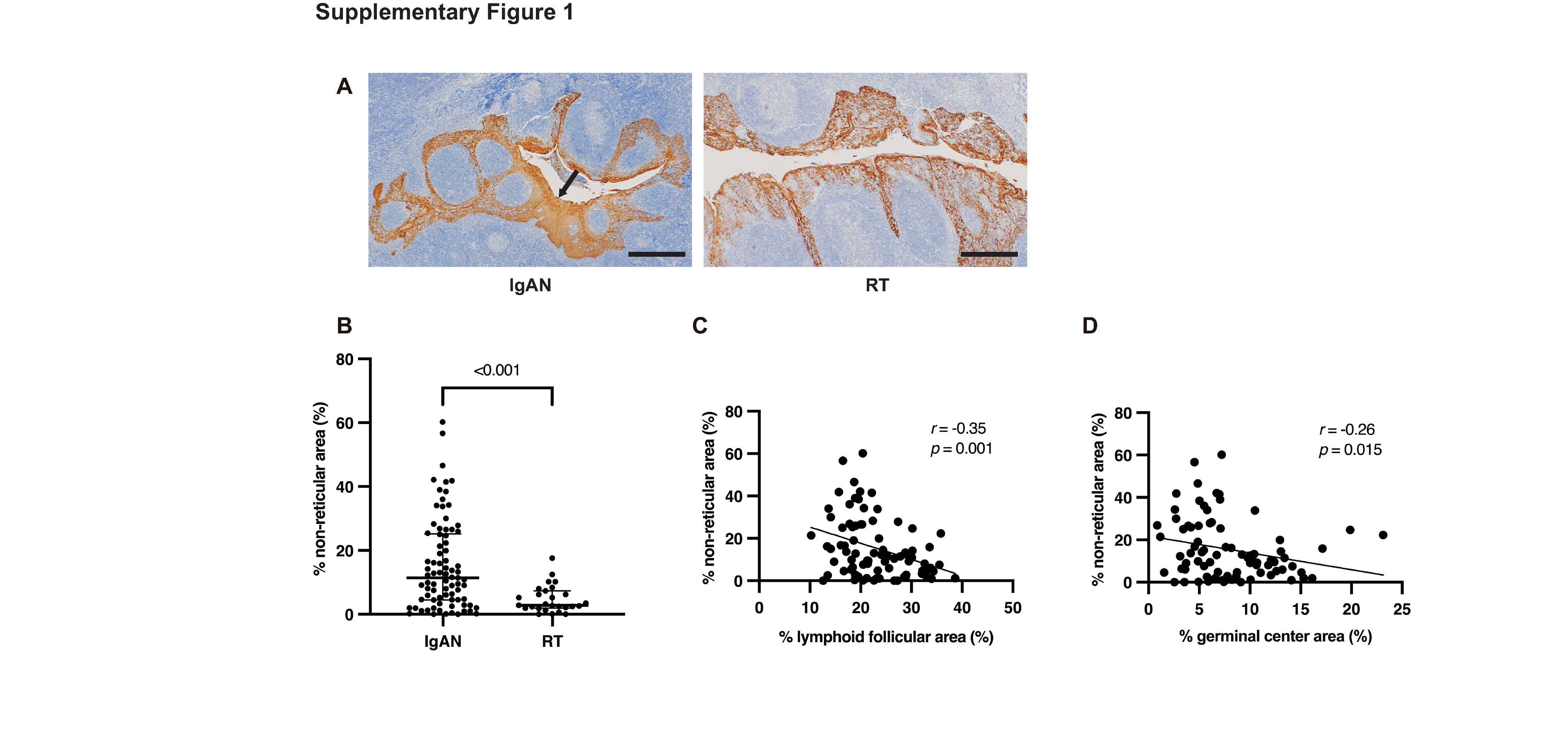

Supplement: S1 Fig — (A) Immunostaining with cytokeratin demonstrates crypt epithelia in the IgAN (right panel) and RT (left panel) tonsils. Lack of reticulation (arrow) in crypt epithelia is more frequently observed in IgAN tonsils than in RT tonsils. Magnification: 40×; scale: 500 μm. (B) The median percentage of non-reticular area in the crypt epithelia (% non-reticular area) was higher in the IgAN tonsils than in the RT tonsils (P < 0.001). (C and D) % non-reticular area was inversely correlated with % lymphoid follicular area (C) (r = − 0.35, P = 0.001) and % germinal center area (D) (r = − 0.26, P = 0.015). (TIF) [file pone.0301853.s001.tif]
